# Differential Effects of the Betablockers Carvedilol, Metoprolol and Bisoprolol on Cardiac K_v_4.3 (I_to_) Channel Isoforms

**DOI:** 10.3390/ijms241813842

**Published:** 2023-09-08

**Authors:** Ann-Kathrin Rahm, Juline Hackbarth, Mara E. Müller, Julia Pfeiffer, Heike Gampp, Finn Petersenn, Rasmus Rivinius, Norbert Frey, Patrick Lugenbiel, Dierk Thomas

**Affiliations:** 1 Heidelberg Center for Heart Rhythm Disorders, Heidelberg University Hospital, 69120 Heidelberg, Germanymara.mueller@med.uni-heidelberg.de (M.E.M.); rasmus.rivinius@med.uni-heidelberg.de (R.R.); patrick.lugenbiel@med.uni-heidelberg.de (P.L.);; 2 Department of Cardiology, Heidelberg University Hospital, 69120 Heidelberg, Germany; 3 DZHK (German Center for Cardiovascular Research), Partner Site Heidelberg/Mannheim, 69120 Heidelberg, Germany

**Keywords:** K_v_4.3, I_to_, betablocker, antiarrhythmic effects, heart failure

## Abstract

Cardiac K_v_4.3 channels contribute to the transient outward K^+^ current, I_to_, during early repolarization of the cardiac action potential. Two different isoforms of K_v_4.3 are present in the human ventricle and exhibit differential remodeling in heart failure (HF). Cardioselective betablockers are a cornerstone of HF with reduced ejection fraction therapy as well as ventricular arrhythmia treatment. In this study we examined pharmacological effects of betablockers on both K_v_4.3 isoforms to explore their potential for isoform-specific therapy. K_v_4.3 isoforms were expressed in Xenopus *laevis* oocytes and incubated with the respective betablockers. Dose-dependency and biophysical characteristics were examined. HEK 293T-cells were transfected with the two K_v_4.3 isoforms and analyzed with Western blots. Carvedilol (100 µM) blocked K_v_4.3 L by 77 ± 2% and K_v_4.3 S by 67 ± 6%, respectively. Metoprolol (100 µM) was less effective with inhibition of 37 ± 3% (K_v_4.3 L) and 35 ± 4% (K_v_4.3 S). Bisoprolol showed no inhibitory effect. Current reduction was not caused by changes in K_v_4.3 protein expression. Carvedilol inhibited K_v_4.3 channels at physiologically relevant concentrations, affecting both isoforms. Metoprolol showed a weaker blocking effect and bisoprolol did not exert an effect on K_v_4.3. Blockade of repolarizing K_v_4.3 channels by carvedilol and metoprolol extend their pharmacological mechanism of action, potentially contributing beneficial antiarrhythmic effects in normal and failing hearts.

## 1. Introduction

The cardiac K_v_4.3 channel is the main carrier of the transient outward potassium current (I_to_) during the early repolarization phase of the cardiac action potential [1]. Changes in expression and function of this channel are associated with cardiac conditions such as Brugada syndrome, atrial fibrillation, and early repolarization syndrome. Moreover, K_v_4.3 expression changes have been reported after myocardial infarction and heart failure (HF) [2,3,4]. A consistent electrophysiological feature in HF is a prolongation of the ventricular action potential [5,6]. In animal HF models, as well as in human cardiac myocytes, a reduction of the transient outward potassium current in the early repolarization phase mediated by K_v_4.3 was observed [7,8,9,10,11]. The cardiac K_v_4.3 channel, which is encoded by the *KCND3* gene, is activated voltage dependently. There are two different isoforms that result from the alternative splicing of exon 6. The longer K_v_4.3 L variant and the shorter isoform K_v_4.3 S differ in a 19 amino acid long sequence at the intracellular C-terminus [12,13]. In patients with dilated cardiomyopathy expression levels of K_v_4.3, isoforms are differentially expressed: K_v_4.3 L is upregulated, whereas K_v_4.3 S is downregulated. As a result, the long isoform is predominantly expressed in the insufficient heart [4]. 

Betablockers constitute a cornerstone of pharmacological HF therapy [14]. Metoprolol, bisoprolol and carvedilol reduced mortality in HF patients [15,16,17]. Betablockers attenuate the proarrhythmic effect of catecholamines, which are pathologically elevated in HF. They exert negative ino-, chrono-, dromo-, and bathmotropic effects and directly affect ventricular remodeling through alterations in expression and function of ion channels. In particular, carvedilol interacts with cardiac ion channels, including human ether-a-go-go-related gen (hERG) K^+^ channels and two-pore-domain potassium channels (K_2P_) [18,19,20,21,22]. 

The aim of this study was to examine differential effects of carvedilol, bisoprolol, and metoprolol on K_v_4.3 isoforms to provide the basis for isoform-specific HF drug therapy.

## 2. Results

### 2.1. Effects of Carvedilol on the Function of K_v_4.3 Channel Isoforms 

**Figure 1:**
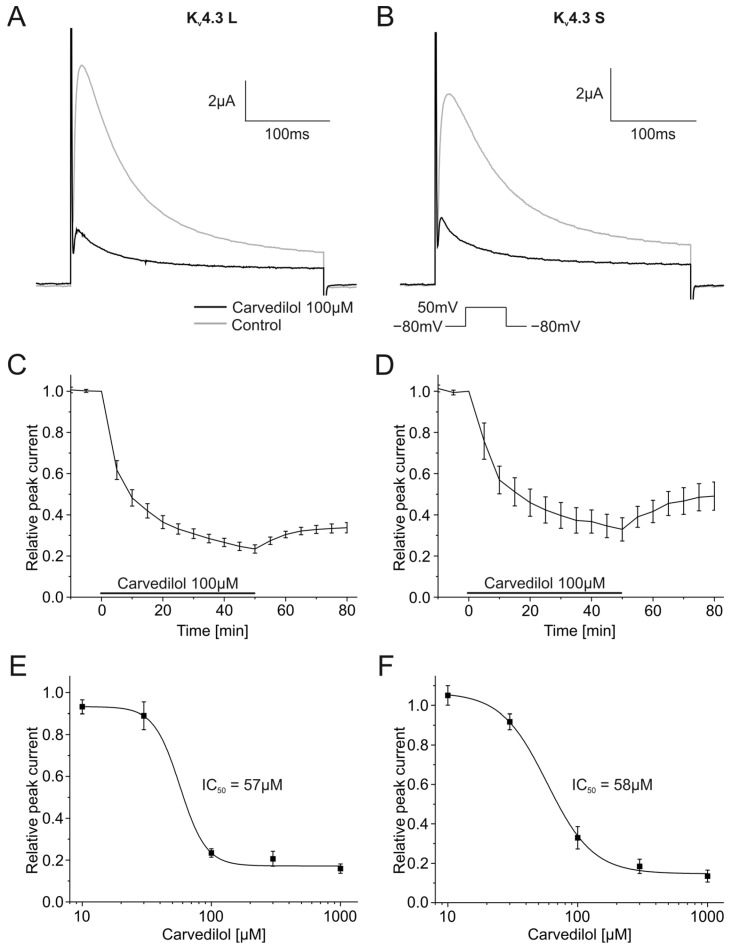
Effects of carvedilol (100 µM) on currents produced by the K_v_4.3 isoform. (**A**,**B**) Representative current traces of K_v_4.3 L (**A**) and K_v_4.3 S (**B**) prior to (gray) and after (black) carvedilol application (50 min). (**C**,**D**) Relative peak current of K_v_4.3 L (*n* = 8) (**C**) and K_v_4.3 S (*n* = 8) (**D**) before, during and after the application 100 µM carvedilol. (**E**,**F**) Concentration-response curves for K_v_4.3 L (*n* = 8–12) (IC_50_ = 57.1 ± 12.6 µM) (**E**) and K_v_4.3 S (*n* = 8–13) (IC_50_ = 58.2 ± 6.2 µM) (**F**).

Carvedilol caused time-dependent inhibition of the K_v_4.3 peak current. Both isoforms were similarly affected. Maximum inhibitory effects observed after 50 min yielded a current block of 76.6 ± 2.0% (K_v_4.3 L, *n* = 8, *p* < 0.001) and 67.1 ± 5.7% (K_v_4.3 S, *n* = 8, *p* < 0.001). K_v_4.3 currents before and after application of carvedilol, as well as the development of the block, are shown in Figure 1A–D. 

The inhibition of K_v_4.3 L and K_v_4.3 S currents was partially reversible. The peak current returned to 33.8 ± 2.6% for the L isoform, and 49.1 ± 6.8% for the S isoform after 30 min wash out. Blockade was concentration dependent with IC_50_ values of 57.1 ± 12.6 µM (K_v_4.3 L, *n* = 8–12) and 58.2 ± 6.2 µM (K_v_4.3 S, *n* = 8–13) (Figure 1E,F). 

### 2.2. Biophysical Characteristics of K_v_4.3 Inhibition by Carvedilol 

**Figure 2:**
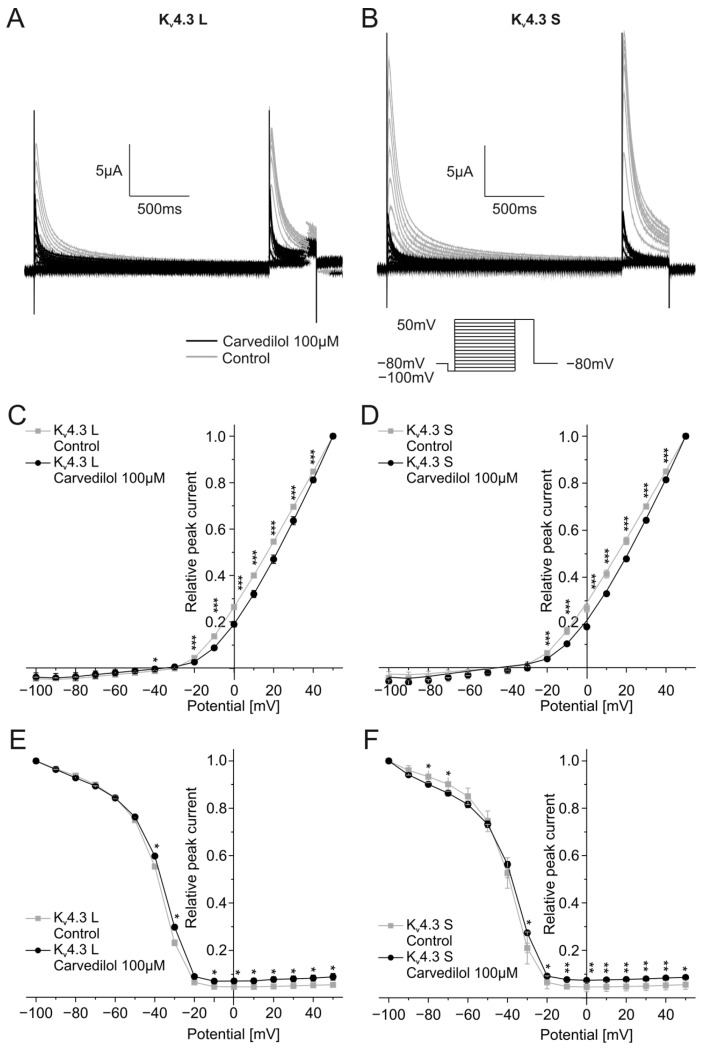
Effects of carvedilol (100 µM) on activation and inactivation of the K_v_4.3 isoforms. (**A**,**B**) Representative current traces of K_v_4.3 L (**A**) and S (**B**) evoked by the indicated voltage protocol prior to (gray) and after (black) carvedilol application (50 min). (**C**,**D**) I-V-plots for activation of K_v_4.3 L (**C**) and K_v_4.3 S (**D**) (*n* = 11). (**E**,**F**) I-V-plots for inactivation of K_v_4.3 L (**E**) and K_v_4.3 S (**F**) (*n* = 11). * *p* < 0.05, ** *p* < 0.01, *** *p* < 0.001.

Activation and inactivation kinetics were assessed by applying depolarizing steps from −100 mV to +50 mV (2000 ms, 10 mV increments) from a holding potential of −80 mV. There was a return pulse to +50 mV after the first voltage step. The double-step voltage protocol as well as typical current traces in the absence and the presence of 100 µM carvedilol are depicted in Figure 2A,B. 

To examine the effects of carvedilol on K_v_4.3 channel activation, peak currents measured during the first part of the voltage protocol were normalized to the peak current of the last trace and then plotted against the test pulse voltage. Application of carvedilol led to a shift of the half-maximal activation voltages of both K_v_4.3 isoforms towards more positive voltages. The half-maximal activation voltage in the presence of carvedilol (K_v_4.3 L: V_1/2_ = 31.3 ± 4.0 mV, *n* = 11, *p* < 0.0001, K_v_4.3 S: V_1/2_ = 32.0 ± 3.1 mV, *n* = 11, *p* < 0.0001) differed significantly from the measurements under control conditions (K_v_4.3 L: V_1/2_ = 22.6 ± 2.3 mV, *n* = 11, K_v_4.3 S: V_1/2_ = 22.4 ± 1.6 mV, *n* = 11) (Figure 2C,D). 

The current peak evoked by the second voltage step reflects channel inactivation. Inactivation of the two K_v_4.3 isoforms was analyzed by plotting the peak current during the second step of the voltage protocol against the respective test pulse potential. Half-maximal inactivation voltages after application of 100 µM carvedilol were numerically more positive compared to control conditions without reaching statistical significance. K_v_4.3 L half-maximal inactivation voltage was −39.5 ± 1.3 mV under control conditions and −38.3 ± 1.3 mV after incubation with carvedilol (*n* = 11, *p* = 0.016), whereas values obtained with K_v_4.3 S yielded −40.2 ± 2.1 mV under control conditions and −39.5 ± 1.6 mV after carvedilol administration (*n* = 11, *p* = 0.398) (Figure 2E,F).

**Figure 3:**
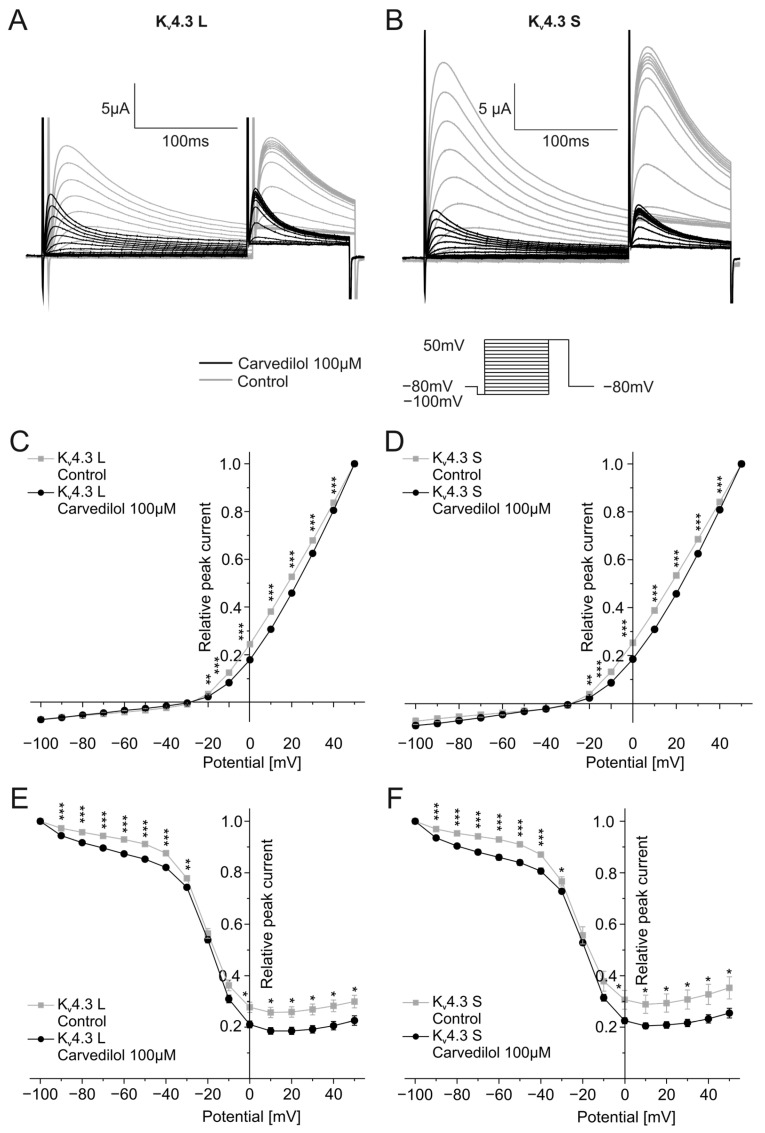
Effects of carvedilol (100 µM) on the activation and inactivation of the two K_v_4.3 isoforms with short depolarizing steps. (**A**,**B**) Representative current traces of K_v_4.3 L (**A**) and S (**B**) induced by the indicated voltage protocol prior to (gray) and after (black) carvedilol application (50 min). (**C**,**D**) I-V plots for activation of K_v_4.3 L (**C**) and K_v_4.3 S (**D**) (*n* = 11). (**E**,**F**) I-V-plots for inactivation of K_v_4.3 L (**E**) and K_v_4.3 S (**F**) (*n* = 11). * *p* < 0.05, ** *p* < 0.01, *** *p* < 0.001.

To reflect ventricular tachyarrhythmias more closely with shorter cycle lengths, activation and inactivation kinetics were additionally analyzed by applying shorter depolarizing steps of 200 ms (Figure 3). Similar to the protocol described above, steps ranging from −100 mV to +50 mV were used from a holding potential of −80 mV with increments of 10 mV. Typical current traces are depicted in Figure 3A,B. Activation and inactivation voltages were analyzed as described before. Half-maximal activation voltages were again significantly different between control conditions (K_v_4.3 L: V_1/2_ = 25.1 ± 2.1 mV, K_v_4.3 S: V_1/2_ = 24.6 ± 2.8 mV) and after 50 min incubation with 100 µM carvedilol (K_v_4.3 L: V_1/2_ = 34.8 ± 4.8 mV, *n* = 11, *p* < 0.0001, K_v_4.3 S: V_1/2_ = 39.9 ± 15.2 mV, *n* = 11, *p* = 0.010) (Figure 3C,D). Furthermore, half-maximal inactivation voltages were slightly more positive after carvedilol administration compared to drug-free conditions, resembling findings obtained with longer voltage pulses. The difference was not significant for both isoforms: The half-maximal inactivation voltage of K_v_4.3 was −22.5 ± 1.6 mV (control) and −21.7 ± 1.2 mV after incubation with carvedilol (*n* = 11, *p* = 0.123). For K_v_4.3 S, values of −23.6 ± 3.1 mV under control conditions and −22.7 ± 1.7 mV after treatment with carvedilol (*n* = 11, *p* = 0.464) were calculated (Figure 3E,F). 

**Figure 4:**
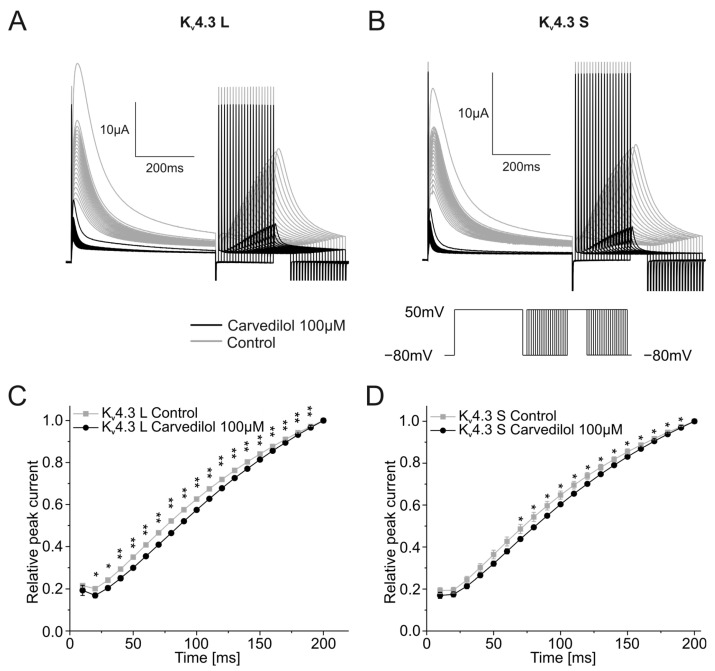
Effects of carvedilol (100 µM) on the recovery from inactivation of the two K_v_4.3 isoforms. (**A**,**B**) Representative K_v_4.3 L (**A**) and K_v_4.3 S (**B**) current traces of induced by the indicated voltage protocol prior to (gray) and after (black) carvedilol application (50 min). (**C**,**D**) Recovery from inactivation curves were calculated by plotting peak current amplitudes against the duration of the preceding repolarizing step for K_v_4.3 L (**C**) and K_v_4.3 S (**D**) (*n* = 10). * *p* < 0.05, ** *p* < 0.01.

Next, the recovery from K_v_4.3 inactivation was assessed using a multistep protocol. After a depolarization step from a holding potential of −80 mV to +50 mV for 500 ms, a return pulse to the −80 mV holding potential was applied with durations ranging from 10 to 200 ms in 10 ms increments. The variable duration allowed the channel to recover from inactivation and was then followed by a 250 ms long depolarizing step from −80 mV to +50 mV. The peak currents recorded during the final variable voltage step were normalized to the highest peak current and then plotted against the respective duration of the preceding step. Representative traces are depicted in Figure 4A,B. Relative peak currents normalized to the highest peak current after the longest duration of the −80 mV holding potentials (200 ms) did not differ between control measurements and recordings after 100 µM carvedilol activation for 50 min. Time constants of recovery from inactivation were calculated after applying a single exponential fit (Figure 4C,D). Significant differences between time constants under control conditions (τ = 267.0 ± 25.8 ms) and after 50 min incubation with carvedilol (τ = 505.2 ± 57.4 ms, *n* = 10, *p* = 0.002) were observed with K_v_4.3 L. For K_v_4.3 S, the increase of the time constant was not significant. Time constants of 229.1 ± 45.3 ms under control conditions and 295.9 ± 20.2 ms in the presence of carvedilol were obtained (*n* = 10, *p* = 0.094).

**Figure 5:**
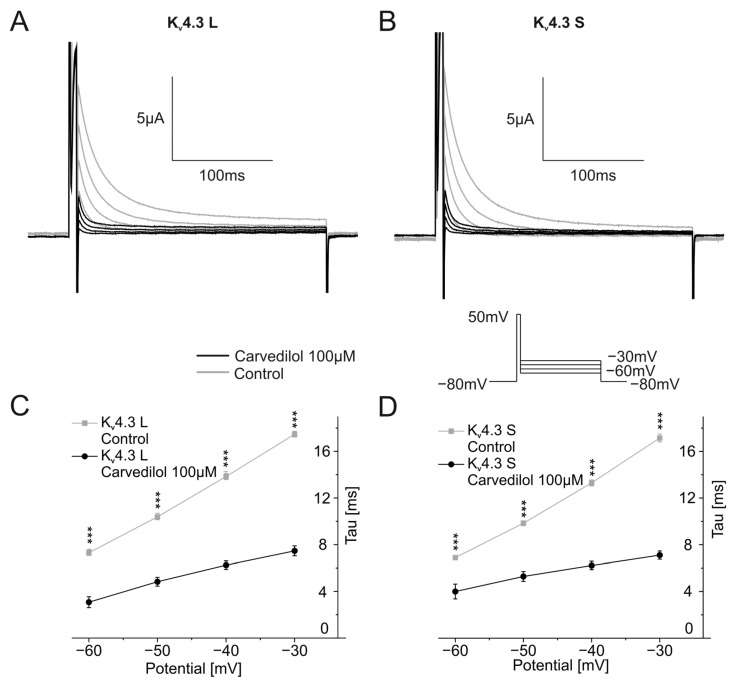
Effects of carvedilol (100 µM) on deactivation of K_v_4.3 isoforms. (**A**,**B**) Representative current traces of K_v_4.3 L (**A**) and K_v_4.3 S (**B**) induced by indicated voltage protocol prior to (gray) and after (black carvedilol application (50 min)). (**C**,**D**) Deactivation time constants for K_v_4.3 L (**C**) and K_v_4.3 S (**D**) (*n* = 11). *** *p* < 0.001.

Finally, channel deactivation was analyzed by briefly depolarizing the oocyte from −80 mV to +50 mV for 7.5 ms, followed by second 250 ms-long voltage steps ranging from −60 mV to −30 mV in 10 mV increments. Representative traces are shown in Figure 5A,B. Time constants of deactivation were calculated by applying single exponential fits to deactivating currents of the second part of the protocol and were then plotted against the voltage of the second pulse of the voltage protocol. Time constants showed a linear behavior. For control conditions, as well as for the measurement after incubation with 100 µM carvedilol, channel deactivation accelerated with more negative membrane potentials (Figure 5C,D). Carvedilol caused further acceleration of channel deactivation compared to control conditions. Time constants differed significantly between the control measurements and after betablocker incubation for both channel isoforms (*n* = 11, *p* < 0.001) (Figure 5C,D).

### 2.3. Effects of Bisoprolol on K_v_4.3 Channel Isoforms

**Figure 6:**
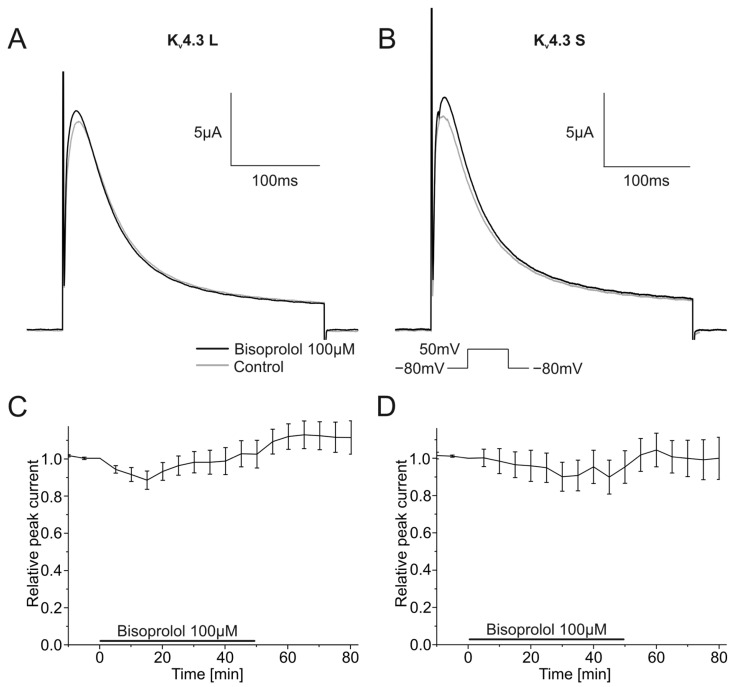
Effects of bisoprolol (100 µM) on K_v_4.3 isoforms. (**A**,**B**) Representative K_v_4.3 L (**A**) and K_v_4.3 S (**B**) current traces of prior to (gray) and after (black) bisoprolol application (50 min). (**C**,**D**) Relative peak current carried by K_v_4.3 L (*n* = 11) (**C**) or K_v_4.3 S (*n* = 9) (**D**) during application of 100 µM bisoprolol.

The impact of the bisoprolol on currents carried by K_v_4.3 isoforms was analyzed as described earlier using a single step depolarizing voltage protocol. Example traces are depicted in Figure 6A,B. Bisoprolol neither affected K_v_4.3 L nor K_v_4.3 S current amplitudes (Figure 6A,B). Peak currents did not change significantly during the 50 min wash-in compared to control measurements for K_v_4.3 L (102.3 ± 7.5%, *n* = 11, *p* = 0.765) or K_v_4.3 S (95.3 ± 8.7%, *n* = 9, *p* = 0.607) (Figure 6C,D). 

### 2.4. Effects of Metoprolol on the Function of K_v_4.3 Channel Isoforms

**Figure 7:**
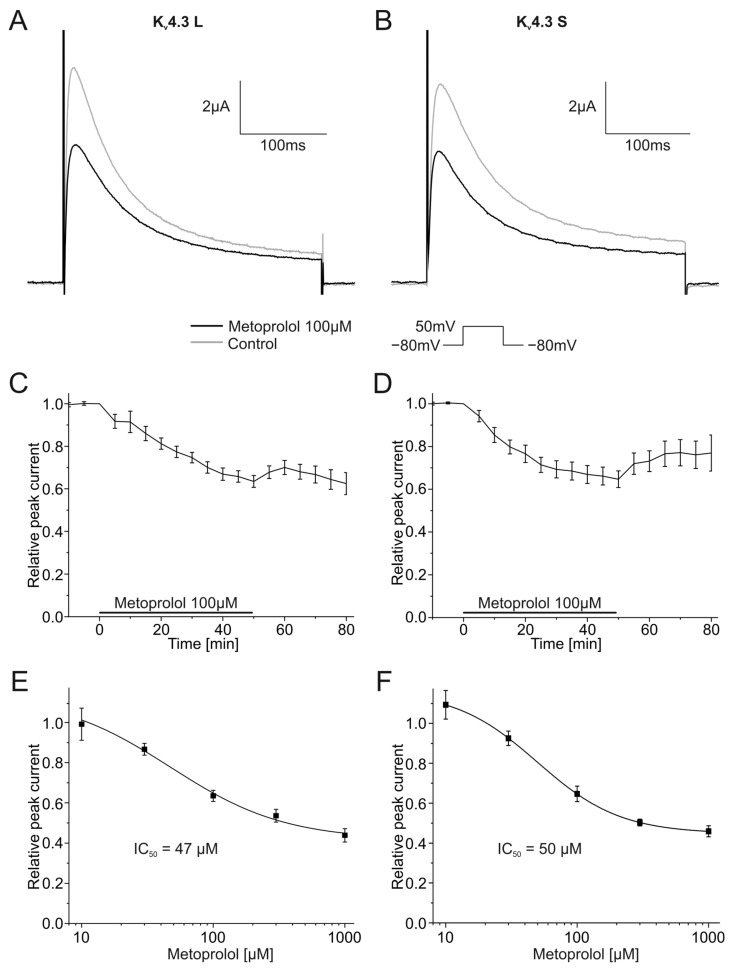
Effects of metoprolol (100 µM) on K_v_4.3 currents. (**A**,**B**) Representative K_v_4.3 L (**A**) and K_v_4.3 S (**B**) current traces of prior to (gray) and after (black) metoprolol application (50 min). (**C**,**D**) Relative peak currents of K_v_4.3 L (*n* = 9) (**C**) and K_v_4.3 S (*n* = 9) (**D**) before, during and after application of 100 µM metoprolol. (**E**,**F**) Concentration-response curves for K_v_4.3 L (*n* = 9–11) (IC_50_ = 47.3 ± 34.2 µM) (**E**) and K_v_4.3 S (*n* = 9–11) (IC_50_ = 49.8 ± 0.7 µM) (**F**).

Pharmacological effects of 100 µM metoprolol on both K_v_4.3 isoforms were similarly assessed. K_v_4.3 L currents were blocked 36.5 ± 2.8% (*n* = 9, *p* < 0.001), and K_v_4.3 S 35.3 ± 3.9% (*n* = 9, *p* < 0.001) during metoprolol application (Figure 7A,B). The degree of current block did not differ between isoforms. Inhibitory effects of metoprolol on K_v_4.3 S appeared to be partially but not significantly reversible; mean peak K_v_4.3 S current normalized to control conditions recovered during 30 min wash-out from 64.7 ± 3.9% in the presence of metoprolol to 76.9 ± 8.5% (*n* = 9) (Figure 7D). No apparent recovery was observed among K_v_4.3 L channels (63.5 ± 2.8% versus 62.5 ± 5.1% after wash out, *n* = 9) (Figure 7C). IC_50_ values for metoprolol blockade yielded 47.3 ± 34.2 µM (*n* = 9–11) for K_v_4.3 L and 49.8 ± 0.7 µM for K_v_4.3 S (*n* = 9–11), respectively (Figure 7E,F). 

### 2.5. Effects of Betablockers on the Expression of K_v_4.3 Isoforms in HEK Cells 

**Figure 8:**
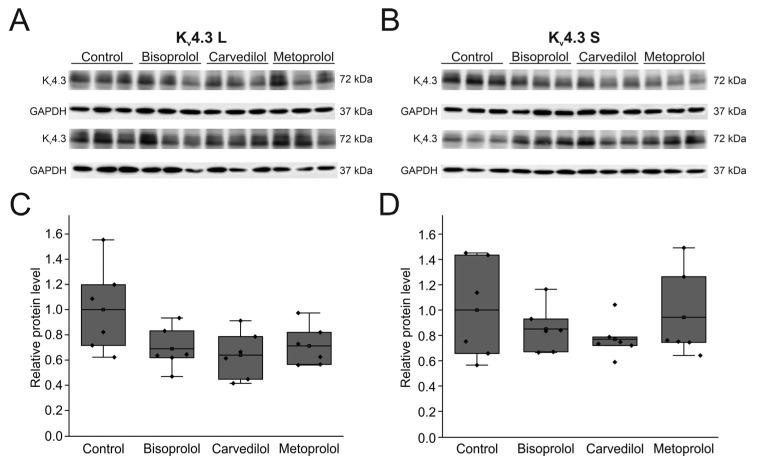
Effects of betablockers on K_v_4.3 isoform expression in HEK-293T cells. (**A**) Representative Western blots after transfection with K_v_4.3 L (**A**) or K_v_4.3 S (**B**) before and after incubation with respective betablockers (100 µM) for 24 h (*n* = 6 each). (**C**,**D**) Protein quantification of K_v_4.3 L (**C**) and K_v_4.3 S (**D**) protein relative to respective controls and normalized to GAPDH.

To study the effects of the betablockers on the expression of Kv4.3 protein, both isoforms were individually expressed in HEK-293T cells (Figure 8A,B). Expression of K_v_4.3 L and K_v_4.3 S whole cell protein was not significantly reduced after incubation with betablockers bisoprolol, carvedilol, or metoprolol, respectively (Figure 8C,D). Protein content relative to control levels were 68.9 ± 6.8% for bisoprolol (*n* = 6, *p* = 0.180), 64.0 ± 7.8% for carvedilol (*n* = 6, *p* = 0.082) and 71.2 ± 6.6% for metoprolol (*n* = 6, *p* = 0.257) for K_v_4.3 L without reaching statistical significance. For the K_v_4.3 S isoform, protein expression relative to control yielded 85.1 ± 7.6% for bisoprolol (*n* = 6, *p* = 1), 77.0 ± 6.1% for carvedilol (*n* = 6, *p* = 1), and 94.2 ± 14.2% for metoprolol (*n* = 6, *p* = 1) without reaching statistical significance. 

## 3. Discussion

Carvedilol and metoprolol significantly blocked two cardiac K_v_4.3 channels isoforms that contribute to repolarization of the cardiac action potential. By contrast, bisoprolol did not affect K_v_4.3 currents, indicating drug-specific actions of carvedilol and metoprolol. 

### 3.1. Mechanisms of Block

The rapid development of the block after application of metoprolol and carvedilol indicates a direct interaction between the drug molecules and the respective K_v_4.3 isoforms as the primary molecular mechanism of action. This assumption is reinforced by changes in biophysical characteristics of the K_v_4.3 current after application of carvedilol. It has been shown that pre-pulse duration affects K_v_4.3 inactivation kinetics [23]. Thus, we compared longer (2000 ms) pre-pulses to shorter (200 ms) voltage steps assuming faster heart rates during arrhythmia, revealing rate-independent inhibition of K_v_4.3 L and K_v_4.3 S isoforms by carvedilol. 

K_v_4.3 L is upregulated and K_v_4.3 S is downregulated in HF [4]. Therefore, an I_to_-targeting antiarrhythmic drug therapy should ideally target the K_v_4.3 L isoform. There were no pronounced differences between the effects on K_v_4.3 L and K_v_4.3 S currents for any of the studied betablockers and biophysical parameters. We conclude from these findings that metoprolol and carvedilol cause K_v_4.3 current inhibition of both isoforms via the same molecular mechanism(s). The K_v_4.3 L type isoform differs from the S isoform in a 19 amino acid long sequence at the C-terminal, intracellular end of the channel [12,13]. This portion of the protein harbors a PKC phosphorylation site. We cannot exclude those indirect differential effects of betablockers on K_v_4.3 isoforms via adrenergic signaling pathways under adrenergic stimulation that are beyond scope of this study. 

Bisoprolol and metoprolol counteracted the decreased K_v_4.3 expression and the concomitant reduction of I_to_ in different HF animal models [24,25]. In our in vitro analysis, the incubation of transfected HEK cells with the respective betablockers did not affect expression levels of the respective K_v_4.3 isoforms. It should be noted that HEK-293T cells endogenously express alpha-1 adrenoreceptors [26], and even potentially possible adrenoreceptor-blocking effects did not change expression levels. 

### 3.2. Clinical Significance

Betablockers bisoprolol, carvedilol, and metoprolol exert a class effect during treatment of HF patients with reduced ejection fraction, with no apparent evidence for the superiority of any single agent over the others [14,27]. However, additional inhibition of potassium channels may suppress cardiac arrhythmias through prolongation of the action potential and by preventing electrical reentry. Multi-channel blocking effects of carvedilol may contribute to these antiarrhythmic effects [18,19,20,21,22]. Indeed, carvedilol was superior to metoprolol in small studies in reducing ICD therapies for ventricular arrhythmias [28] and in avoiding inappropriate ICD therapies [29]. 

Inhibition of K_v_4.3 channels by carvedilol or to lesser extent metoprolol could thus exert beneficial antiarrhythmic effects. The drug concentrations used in our study are apparently higher when compared to the maximum therapeutic plasma concentrations: IC_50_ values of 57.1 ± 12.6 µM (L isoform) and 58.2 ± 6.2 µM (S isoform) were obtained for carvedilol. During therapeutic application of carvedilol, maximum plasma concentrations ranging from 0.1–0.6 µM were measured [20] (Table S1 in Supplementary Material). However, compared to mammalian cells, the concentrations for pharmacological ion channel inhibition in Xenopus *laevis* oocytes tend to be about 5 to 10 times higher [30], indicating that carvedilol effects observed here may be physiologically relevant during drug use in humans. 

### 3.3. Potential Limitations

This study focused on acute, direct effects of betablockers on K_v_4.3 channels. Potential pharmacological effects on other subunits of I_to_ such as K_chip2_ were not analyzed. In addition, other mechanisms beyond direct channel binding were not assessed and need to be investigated in future studies, and cardiac cell lines must be modified to assess isoform specific K_v_4.3 characteristics [31,32]. In contrast to human ventricular cardiomyocytes in heart failure [4,33], differential K_v_4.3 isoform expression and remodeling has not been assessed in cardiac cell lines so far. Finally, clinical consequences and the potential differential effect on arrhythmias would have to be investigated in clinical trials with head-to head comparisons. 

## 4. Materials and Methods

### 4.1. Drugs

Bisoprolol was dissolved in 87% distilled water and 13% dimethyl sulfoxide, carvedilol in 24% distilled water and 76% dimethyl sulfoxide, and metoprolol in distilled water. The betablockers were stored as 100 mM stock solutions for electrophysiological measurements, and as 10 mM stock solutions for the experiments involving HEK cells at room temperature. For the experiments, stock solutions were diluted to the required concentrations. 

### 4.2. Animal Handling and Ethics Statement 

Animal studies were performed in compliance with the Guide for the Care and Use of Laboratory Animals, as approved and published by the U.S. National Institutes of Health (NIH publication No. 85–23, revised 1985) as well as the current version of the German Law on the Protection of Animals. The investigation conforms to the Directive 2010/63/EU of the European parliament. Surgical procedures on female Xenopus *laevis* frogs were institutionally approved (35-9185.81/G-270/17) and performed as previously reported [34].

### 4.3. Expression of K_v_4.3 Channel Isoforms in Xenopus Laevis Oocytes 

DNA encoding K_v_4.3 isoforms L and S were introduced into DH5-α bacteria with the help of the plasmid vector pMAX^−^. Plasmid DNA was then isolated using the QIAprep Spin Miniprep Kit (Qiagen, Hilden, Germany) and linearized using PmeI. Next, the DNA was transcribed using T7 DNA polymerase and the mMessage mMachine Kit (Ambion, Austin, TX, USA). The concentration of the transcribed RNA was determined using NanoDrop 2000 (Thermo Fisher Scientific, Waltham, MA, USA). The RNA was then injected into stages V and VI defolliculated Xenopus *laevis* oocytes using a Nanoinjector (Nanoject II, H. Saur, Reutlingen, Germanys). The injected volume was 46 nl and the concentration of injected cRNA was 10 ng. The electrophysiological measurements were carried out 2–3 days after the injection. 

### 4.4. Expression of K_v_4.3 Channel Isoforms in HEK Cell Line 

Human embryonic kidney (HEK-293T) cells were cultured at 37 °C with 5% CO_2_. HEK cells were transfected with the 1 µg DNA of K_v_4.3 L and S per well using the Lipofectamine ^TM^3000 Transfection reagent (Invitrogen, Thermo Fisher Scientific Inc. Carlsbad, CA, USA). Twenty-four hours after transfection, the cells were incubated with 10 µM of the different betablockers for another 24 h. 

### 4.5. Voltage-Clamp Electrophysiology 

Two to three days after RNA injection, the electrophysiological measurements were performed using the two-voltage electrode clamp technique as described before [35]. Currents were recorded using an Oocyte Clamp amplifier (Warner OC-725A, Warner Instruments, Hamden, CT, USA) and Pclamp software version 8.2 (Axon Instruments, Foster City, CA, USA). Data were sampled at 2 kHz and filtered at 1 kHz. Voltage clamp electrodes were pulled from glass capillaries (GB100F-10, Science Products GmbH, Hofheim, Germany) using a micropipette puller (P-1000 Next Generation Micropipette Puller, Sutter Instrument, Novato, CA, USA) filled with 3 M of KCl solution; the electrodes hat tip resistances were 5–10 MΩ. The standard extracellular bath solution contained 96 mM NaCl, 4 mM KCl, 1.1 mM CaCl_2_, 1 mM MgCl_2_ und 5 mM HEPES and was adjusted to pH 7.4 with NaOH. Before the measurements, the oocytes were preincubated in this solution for at least 20 min. The experiments were carried out under steady gravity-driven perfusion at room temperature. 

K_v_4.3 currents were induced with a single depolarizing voltage step from a holding potential of −80 mV to +50 mV for 250 ms. To evaluate the effect of 100 µM carvedilol on K_v_4.3 current, oocytes were first treated with the betablocker for 50 min, followed by washout with a 4 mM K^+^ solution to assess reversibility. Values of measured peak amplitudes were normalized to the value of the peak amplitude obtained during the last measurement prior to drug application. 

### 4.6. Western Blots

For Western Blot studies, HEK cells were lysed in a radioimmunoprecipitation (RIPA) buffer consisting of 20 mM Tris-HCl, 0.5% NP-40, 0.5% sodium-deoxycholate, 150 nM NaCl, 1 mM EDTA, 1 mM Na_3_VO_4_, 1 mM NaF and inhibitors proteases (CompleteMini, Roche Applied Science, Indianapolis, IN, USA). Those lysed cells were incubated on ice for 20 min and then centrifugated for 30 min at 14,000× *g* and 4 °C. The supernatants were then collected, and the protein concentration was determined using the bicinchoninic acid (BCA) protein assay (Thermo Scientific, Rockford, IL, USA). The proteins were then diluted to equal concentrations with water. Next, equal amounts of proteins were separated on a 10% SDS polyacrylamide gel and then transferred onto polyvinylidene difluoride membranes (AmershamTM Protran R Western blotting membranes, nitrocellulose, Cytiva, Marlborough, MA, USA). After being blocked with 5% milk in PBST for 2 h at room temperature, those membranes were incubated with primary antibodies directed against K_v_4.3 (1:1000 dilution, #APC-017, rabbit, Alomone Lab, Jerusalem, Israel) at 4 °C overnight. As a control, the respective control peptides for K_v_4.3, supplied by the company, were used (BLP-PC017, Alomone Lab, Jerusalem, Israel). A secondary antibody donkey-anti-rabbit (1:3000 dilution, ab6802, Abcam, Cambridge, UK) was used. Signals were developed using the Azure 600 Ultimate Western Imaging (Azure Biosystems, Dublin, CA, USA) and ECL^TM^ Select Western Blotting Detection Reagent (Cytiva, Marlborough, MA, USA). After removal of the primary and secondary antibodies (ReBlot Strong Stripping Solution, Merck, Germany), the membranes were incubated in anti-GAPDH primary antibodies (1:20,000, ab181602, Abcam, Cambridge, UK) and the corresponding secondary donkey-anti-rabbit antibody (1:3000 dilution, ab6802, Abcam, Cambridge, UK). Optical density was quantified using ImageJ 1.50i Software (National Institutes of Health, Bethesda, MD, USA). 

### 4.7. Data Analysis and Statistics 

Data was analyzed using Origin2022 software (OriginLab, Northhampton, MA, USA) and Microsoft Excel software 2021 (Microsoft, Redmond, WA, USA). Data are expressed as mean ± standard error of the mean (SEM). The concentration response curve was fitted with a Hill1 function (y = START + ((END − START) x^n^)/(k^n^ + x^n^)). Curves for activation and inactivation were fitted with a Boltzmann function (y = A2 + (A1 − A2)/(1 + exp((x − x0)/dx))). Current traces for the deactivation measurements were fitted with a one-phase exponential decay function with time constant parameter (ExpDec1) (y = y_0_ + Ae^−x/t^). Curves for the recovery measurements were fitted with a one-phase exponential association equation (ExpAssoc1) (y = Yb + A × (1 − e^−(x−TD)/Tau)^). Kolmogorov–Smirnov tests were used to confirm normal distribution of the data. To test the statistical significance, paired Student´s *t*-tests were applied for all the electrophysiological measurements. For the statistical analysis of the Western Blots, ANOVA tests were used. *p* < 0.05 was considered statistically significant. 

## 5. Conclusions

Widely used cardioselective betablockers exert differential effects on cardiac repolarizing K_v_4.3 channels underlying the I_to_ current. Carvedilol has a strong inhibitory effect on the K_v_4.3 isoforms, whereas metoprolol was less effective. Concentrations required for blockade were within upper physiological ranges. Bisoprolol did not have any effect on K_v_4.3 currents. Specific electropharmacological actions of carvedilol and metoprolol may be considered when choosing betablockers for HF therapy. 

## Data Availability

Raw data may be acquired from the corresponding author on reasonable request.

## Supplementary Materials

The following supporting information can be downloaded at: https://www.mdpi.com/article/10.3390/ijms241813842/s1. References [36,37,38,39,40,41,42,43,44,45,46,47,48,49,50,51,52,53,54,55,56,57] are cited in the Supplementary Materials.
