# Supplementary material for: Differential Effects of the Betablockers Carvedilol, Metoprolol and Bisoprolol on Cardiac Kv4.3 (Ito) Channel Isoforms
Source: Int J Mol Sci. 2023 Sep 8;24(18):13842. doi: 10.3390/ijms241813842 (PMC10530285; doi:10.3390/ijms241813842)
Supplement: Supplementary file 1 [file ijms-24-13842-s001.zip › Supplemental Material v2.pdf]

Differential Effects of the Betablockers Carvedilol, Metoprolol and Bisoprolol on Cardiac $K_v4.3$ (I_{to}) Channel Isoforms

Ann-Kathrin Rahm [1,2,3,*,†], Juline Hackbarth [1,2,†], Mara E. Müller [1,2,3], Julia Pfeiffer [1,2], Heike Gampp [1,2], Finn Petersenn [1,2], Rasmus Rivinius [1,2], Norbert Frey [1,2,3], Patrick Lugenbiel [1,2,3] and Dierk Thomas [1,2,3]

[1] Heidelberg Center for Heart Rhythm Disorders, Heidelberg University Hospital, 69120 Heidelberg, Germany; mara.mueller@med.uni-heidelberg.de (M.E.M.); rasmus.rivinius@med.uni-heidelberg.de (R.R.); patrick.lugenbiel@med.uni-heidelberg.de (P.L.)
[2] Department of Cardiology, Heidelberg University Hospital, 69120 Heidelberg, Germany
[3] DZHK (German Center for Cardiovascular Research), Partner Site Heidelberg/Mannheim, 69120 Heidelberg, Germany
* Correspondence: ann-kathrin.rahm@med.uni-heidelberg.de
† These authors contributed equally to this work.

Table S1 – plasma concentrations of cardioselective betablockers in clinical use

**Carvedilol**<br>Maximum dosage: 25mg single dose, 50mg daily maximum dose<br>Molecular weight: 406.474g/mol			
**Used dose**	c_{max}	**Studied group**	**Reference**
25mg IR (immediate release):<br><br>80mg CR (controlled release):	R -> 149.1nM<br>S -> 63.7nM<br>R + S -> 212.8nM<br><br>R -> 132.4nM<br>S -> 55.8nM<br>R + S -> 188.2nM	Patients with stable, mild, moderate, or severe HF as well as survivors of a recent acute myocardial infarction<br>(*n* = 188)	[36]
25mg	-> 108 (68.7) ng/ml<br>-> 265.7nM	Male patients with chronic HF<br>(*n* = 22)	[37]
12.5mg on Day 1:<br><br>25mg on Day 9:	46.7 (23.3)ng/ml -> 114.9nM<br>104 (58.9)ng/ml -> 255.9nM	Patients with mild to moderate essential hypertension<br>(*n* = 12)	[38]
25mg	-> 53.84 (45.10)ng/ml<br>-> 132,5nM	Volunteers<br>(*n* = 36)	[39]
12.5mg Carvedilol	-> 17.8 ± 11.3ng/ml R<br>-> 8.5 ± 6.4ng/ml S	Healthy subjects<br>(*n* = 12)	[40]
128 mg SR (slow release) carvedilol	-> 77.04 (66.99)ng/ml<br>-> 189.5nM	Healthy male subjects<br>(*n* = 30)	[41]
**Metoprolol**<br>Maximum dosage: 200mg metoprolol tartrate/190mg metoprolol succinate<br>Molecular weight: 684.81g/mol (metoprolol tartrate)			
**Used dose**	c_{max}	**Studied group**	**Reference**
200mg	-> Day 1: 17 (82)ng/ml -> 261.4nM	Healthy male volunteers<br>(*n* = 6)	[42]

	-> Day 8: 247 (167)ng/ml -> 360nM		
2 x 100mg	-> Day 1, Young: 115 ± 24ng/ml -> 167.9nM<br>-> Day 8, Young: 190 ± 20ng/ml -> 277.4nM<br>-> Day 1, Elderly: 106 ± 24ng/ml -> 154.8nM<br>-> Day 8, Elderly: 169 ± 28ng/ml -> 246.8nM	Healthy female volunteers<br>(*n* = 15)	[43]
100mg	-> Control group: 103 (66)ng/ml<br>-> 150.4nM<br>-> Oral contraceptive group: 140 (57)ng/ml<br>-> 204.4nM	Young female volunteers<br>(*n* = 11 for control group, *n* = 12 for oral contraceptive group)	[44]
100mg	-> 210.72 (100.23)ng/ml<br>-> 307.7nM	Healthy volunteers<br>(*n* = 32)	[45]
50mg	-> 52.7 ± 22.0ng/ml<br>-> 77.0nM	Healthy male volunteers<br>(*n* = 12)	[46]
100mg	-> 77.67ng/ml<br>-> 113.4nM	Healthy male volunteers<br>(*n* = 6)	[47]
100mg	-> 101 (54.2)ng/ml<br>-> 147.5nM	Healthy men<br>(*n* = 31)	[48]
100mg	-> 132 ± 40.7ng/ml<br>-> 192.8 nM	Healthy men<br>(*n* = 12)	[49]
50mg	-> 65.1ng/ml<br>-> 95.1nM	Healthy volunteers<br>(*n* = 20)	[50]
**Bisoprolol**<br>Maximum dosage: 10mg, maximum daily dosage of 20mg<br>Molecular weight: 325.443g/mol			
**Used dose**	c_{max}	**Studied group**	**Reference**
10mg	-> 54.3 ± 2.1ng/ml<br>-> 166.8nM	Hyperthyroid patients<br>(*n* = 9)	[51]
10mg	-> 51 (43-63)ng/ml<br>-> 156.7nM	Healthy controls<br>(*n* = 11)	[52]
10mg	-> 32.0 (13.6)ng/ml<br>-> 98.3nM	Healthy male volunteers<br>(*n* = 8)	[53]
5mg	Test product<br>-> 20.71 (4.14)ng/ml<br>-> 63.6nM<br>Reference<br>-> 20.67 (4.03)ng/ml<br>-> 63.5nM	Healthy volunteers<br>(*n* = 18)	[54]
10mg	Test<br>-> 44.2 ± 9.6ng/ml<br>-> 135.6nM<br>Reference<br>-> 43.9 ± 8.3ng/ml<br>-> 134.9 nM	Healthy volunteers<br>(*n* = 26)	[55]
10mg	-> 55.5 ± 6.4ng/ml<br>-> 170.5 nM	Healthy volunteers<br>(*n* = 6)	[56]
10mg	-> 52 ng/ml<br>-> 159.8nM	Healthy subjects<br>(*n* = 8)	[57]
